# Clinicopathological analysis of primary adrenal diffuse large B-cell lymphoma: effectiveness of rituximab-containing chemotherapy including central nervous system prophylaxis

**DOI:** 10.1186/2162-3619-2-19

**Published:** 2013-08-02

**Authors:** Satoshi Ichikawa, Noriko Fukuhara, Ai Inoue, Hiroki Katsushima, Rie Ohba, Yuna Katsuoka, Yasushi Onishi, Joji Yamamoto, Osamu Sasaki, Jun Nomura, Osamu Fukuhara, Kenichi Ishizawa, Ryo Ichinohasama, Hideo Harigae

**Affiliations:** 1 Department of Hematology and Rheumatology, Tohoku University Graduate School of Medicine, 1-1 Seiryo-cho, Sendai 980-8574, Japan; 2 Department of Hematopathology, Tohoku University Graduate School of Medicine, Sendai, Japan; 3 Department of Internal Medicine, Sendai City Hospital, Sendai, Japan; 4 Department of Hematology, Miyagi Prefectural Cancer Center, Sendai, Japan; 5 Department of Internal Medicine, NTT East Tohoku Hospital, Sendai, Japan; 6 Department of Internal Medicine, Sendai Red Cross Hospital, Sendai, Japan; 7 Department of Molecular Hematology/Oncology, Tohoku University Graduate School of Medicine, Sendai, Japan

**Keywords:** Primary adrenal lymphoma, Diffuse large B-cell lymphoma, Adrenal insufficiency, Central nervous system infiltration, Rituximab

## Abstract

**Background:**

Primary adrenal lymphoma (PAL) is an extremely rare subtype of extranodal non-Hodgkin’s lymphoma. Some researchers have reported some of the characteristics of PAL and its association with poor prognosis; however, the clinicopathological features of PAL remain to be elucidated.

**Methods:**

From 2008 to 2011 we experienced seven cases of PAL in our institutions. We retrospectively analyzed the clinical and pathological features of these patients.

**Results:**

The patients ranged in age from 50 to 85 years, with a median of 71 years. The overall male:female ratio was 6:1. All seven patients were diagnosed with diffuse large B-cell lymphoma (DLBCL) pathologically. Bilateral adrenal involvement was confirmed in five patients. The median largest tumor diameter at diagnosis was 58 mm. The Ki-67 index was generally high (>70%). All patients were treated with rituximab-containing chemotherapy, and central nervous system (CNS) prophylaxis was conducted for three patients. One patient with CNS involvement at the time of the diagnosis also received whole-brain radiation. The overall survival rate at two years was 57% (median follow-up; 24.8 months). It is noteworthy that the three patients who received a full course of the rituximab-containing regimen and CNS prophylaxis are currently alive without disease relapse, and that none of the seven patients died due to progression of lymphoma.

**Conclusions:**

Primary adrenal DLBCL can be a clinically aggressive disease entity. Rituximab-containing chemotherapy combined with CNS prophylaxis could be a reasonable option for the treatment of PAL; however, analyses of more PAL cases are needed for the establishment of this strategy.

## Introduction

Malignant lymphoma primarily arising in the adrenal gland, i.e., primary adrenal lymphoma (PAL), is an extremely rare disease and accounts for less than 1% of all non-Hodgkin’s lymphoma. Around 100 cases of PAL have been reported in the English literature to date; with the exception of a few investigational reports based on several cases, most have been single case reports and literature reviews [1-5]. These reports suggested the following clinical and pathological characteristics of PAL: a predominance in elderly males, diffuse large B-cell lymphoma (DLBCL) as the most common histology, frequent bilateral adrenal involvement with insufficiency of adrenal function and a tendency for patients to develop central nervous system (CNS) infiltration. Some researchers have argued that there may be a correlation between PAL and Epstein–Barr virus infection [1,6], while others have reported that tumor cells of primary adrenal DLBCL usually express BCL-2 with a non-germinal center B-cell phenotype [7]. However, the accumulation of more information is needed to further elucidate the clinicopathological features of PAL. PAL has also been reported to generally have a poor prognosis with conventional chemotherapy, such as the CHOP regimen, and the optimal therapy for PAL remains to be established. However, a substantial proportion of cases reported in the literature were treated before the development of rituximab, and it is possible that rituximab would improve the prognosis of primary adrenal DLBCL, as it does of typical DLBCL.

We herein analyzed the clinicopathological features and clinical courses of seven cases of PAL encountered at our institutions in recent years; all were pathologically diagnosed as DLBCL and treated with rituximab-containing chemotherapy.

## Results

### Clinical characteristics

From 2008 to 2011 in our institutions, seven cases were newly diagnosed as primary adrenal DLBCL. We thoroughly reviewed their clinical and pathological findings retrospetctively. The clinical features of the patients are shown in Table 1. The patients ranged in age from 50 to 85 years, with a median of 71 years. The overall male:female ratio was 6:1. Only one patient (Case 5) presented with symptoms of adrenal insufficiency and fever. The adrenal tumor of three cases (Cases 1, 2 and 3) was incidentally found by a CT that was performed to evaluate other underlying diseases, such as a renal tumor, chronic hepatitis and pulmonary fibrosis. The remaining three patients had back pain. B-symptoms (fever, weight loss of more than 10% within six months, or night sweating) were observed in only one patient (Case 5).

**Table 1:** Summary of clinical findings

**Case/Age/Sex**	**Initial presentation**	**Tumor type**	**Biopsy strategy**	**Clinical stage**	**PS**	**IPI**	**CT findings**			**PET findings**		**Laboratory data**			
							**Laterality of adrenal tumor**	**Maximal tumor diameter (mm) [Laterality]**	**Other lymphoma lesions [Maximal diameter (mm)]**	**SUVmax in adrenal tumor**	**SUVmax in adjacent LNs**	**LDH (IU/L)**	**sIL-2R(U/mL)**	**Serum cortisol level at baseline (μg/dL)**	Serum cortisol level after ACTH stimulation* (μg/dL)
1/81/M	Incidentaloma	DLBCL	Laparoscopic adrenalectomy	IE-A	0	L-I	Unilateral	40 [L]	None	11.8	No lesions	372†	565†	13.7	24.8
2/69/M	Incidentaloma	DLBCL	Open adrenalectomy	IE-A	0	L-I	Unilateral	80 [L]	None	19.2	No lesions	376†	1374†	7.2	21.9
3/71/M	Incidentaloma	DLBCL	CT-guided needle biopsy	IIE-A	2	H-I	Bilateral	52 [L]	paraaortic LNs [17]	8.0	4.3	319†	1367†	ND	ND
								33 [R]							
4/62/M	Hemiparesis	DLBCL	CT-guided needle biopsy	IV-A	2	H	Bilateral	60 [L]	Brain	24.5	No lesions	381†	ND	ND	ND
								67 [R]							
5/50/M	Fever, shock Cerebral infarction	DLBCL	CT-guided needle biopsy	IIE-B	4	L-I	Bilateral	57 [L]	paraaortic LNs [17]	22.4	3.5	415†	10468†	7.8	15.7‡
								58 [R]							
6/73/M	Back pain	DLBCL	CT-guided needle biopsy	IIE-A	3	H-I	Bilateral	57 [L]	paraaortic LNs [39]	26.0	14.6	345†	7303†	8.8	11.1‡
								22 [R]							
7/85/F	Back pain	DLBCL	CT-guided needle biopsy	IIE-A	2	H-I	Bilateral	67 [L]	paraaortic LNs [19]	21.5	8.6	529†	7777†	10.8	13.0‡
								39 [R]							

*ACTH* adrenocorticotropic hormone, *CT* computed tomography, *DLBCL* diffuse large B-cell lymphoma, *F* female, *H* high; *H-I* high-intermediate, *IPI* international prognosis index, *L* left, *LDH* serum lactate dehydrogenase, *L-I* low-intermediate, *LNs* lymph nodes, *M* male, *ND* not done, *PET* positron emission tomography, *PS* performance status, *R* right, *sIL-2R* serum soluble interleukin-2 receptor, *SUVmax* maximum standardized uptake value.

*Each serum cortisol level was measured at 60 min after ACTH challenge.

†The value exceeds upper limit of normal. ‡The value is below lower limit of normal.

The mean maximum diameter of the tumor at diagnosis was 58 mm, with a range from 40 to 80 mm. Two patients with unilateral adrenal involvement underwent surgical resection due to an initial suspicion of adrenal carcinoma. On the other hand, the remaining five patients with bilateral adrenal involvement were diagnosed by needle biopsy. Four bilateral cases (Cases 3, 5, 6 and 7) had paraaortic lymph node lesions; all of which were smaller than the adrenal lesions. These patients were stratified to be in a high-risk group by the International Prognosis Index (IPI) [8]. No additional lesions, including mediastinal and CNS lesions, were detected in any cases on CT.

Positron emission tomography (PET) with 2-[^18^F] fluoro-2-deoxy-d-glucose (FDG) was performed in all cases. The uptake of FDG in the adrenal tumor was confirmed in all cases, and in four cases, uptake was also detected in adjacent paraaortic lymph nodes. No other lesions with significant FDG uptake were detected in any of the patients. Uptake foci at the adrenal tumors were revealed in all cases, and the maximum standardized uptake values (SUVmax) in each case were generally high (8.0 – 26.0, median, 21.5). The SUVmax seemed to be higher in the cases with bilateral adrenal infiltration (*n* = 5; median, 22.4) than in the cases with unilateral infiltration (*n* = 2; median, 15.5). In addition, the values of the SUVmax in adjacent lymph nodes were all lower than those in the adrenal tumors. Bone marrow infiltration was not detected in any of the patients.

Apparent CNS infiltration was not confirmed at the time of diagnosis in any of the cases except in Case 4, in which a brain tumor and bilateral adrenal tumors were detected simultaneously. Both the cerebral and adrenal lesions were biopsied and concluded to have the same histology as DLBCL. In Case 5, multiple cerebral infarctions emerged a month before the patient’s presentation to our institutions. This may have been caused by the infiltration of lymphoma cells toward cerebral vessels, although magnetic resonance imaging of the brain and cytology of the cerebrospinal fluid did not detect any infiltration of lymphoma cells.

The serum lactic dehydrogenase (LDH) level was above the normal limits in all cases. The serum level of soluble interleukin-2 receptor (sIL-2R) was also elevated in all six cases in which it was measured. The rapid adrenocorticotropic hormone (ACTH) stimulation test, in which a cortisol level less than 18 – 20 μg/dL at 60 minutes after ACTH challenge is regarded to be a poor response [9], revealed partial adrenal insufficiency in two cases with bilateral adrenal involvement without clinically apparent adrenal insufficiency.

### Pathological analyses

**Figure 1:**
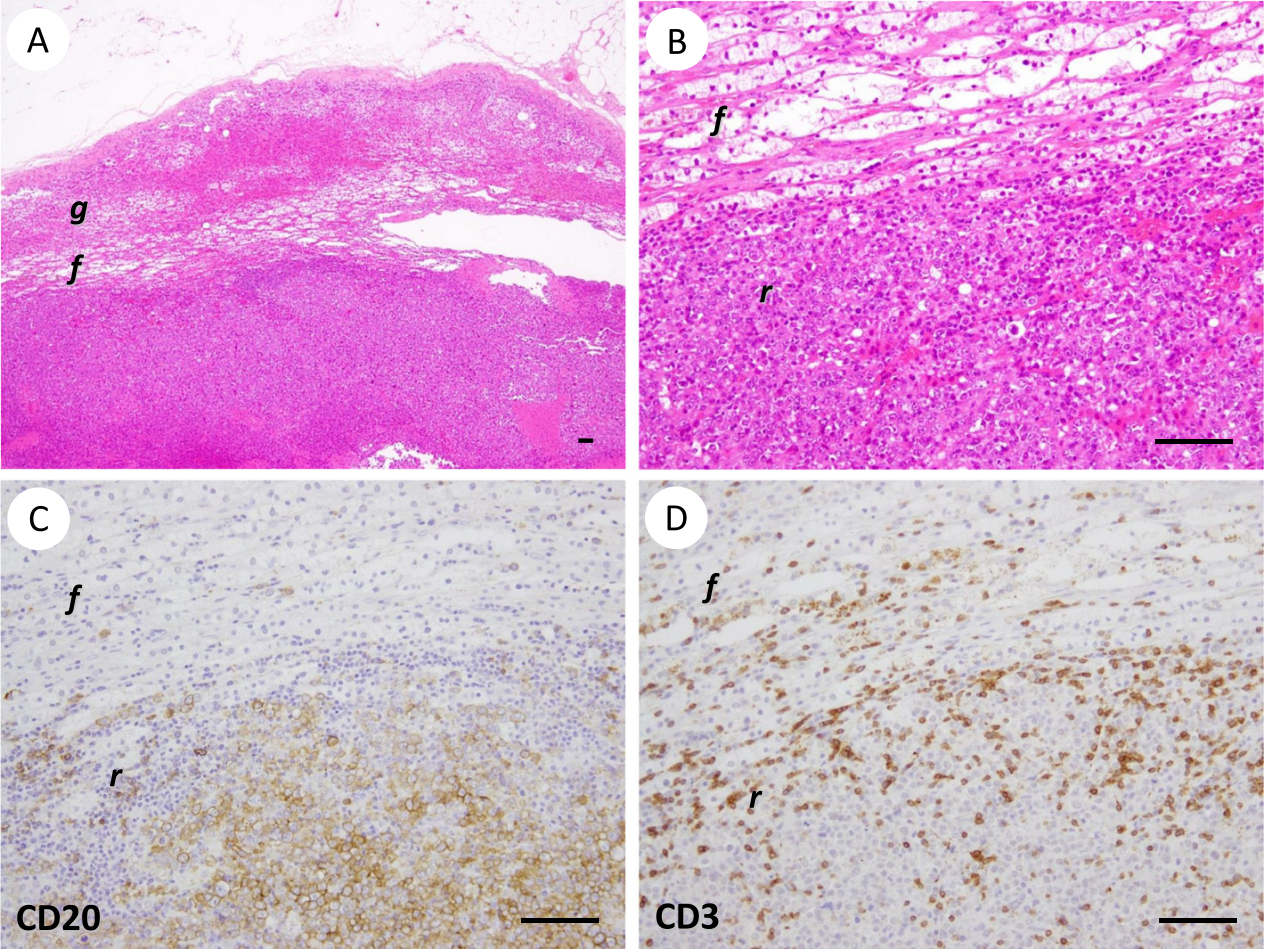
**Representative pathological findings of primary adrenal diffuse large B-cell lymphoma (Case 1).** The black lines in the lower right corner of each figure indicate 100 μm. **(A, B)** The results of hematoxylin and eosin staining. On low-power magnification **(A)**, the lymphoma was located in the zona reticularis (represented by ***r***) of the adrenal cortex. ***f*** and ***g*** represent the zona fasiculata and zona glomerulosa, respectively. High-power magnification **(B)** showed medium- to large-sized atypical lymphoid cells with dispersed chromatin. **(C)** Immunohistochemical staining for CD20 showed that the large lymphoma cells were positive for CD20. **(D)** Immunohistochemical staining for CD3 showed reactivity in small lymphocytes, but not in large lymphoma cells.

The pathological and immunohistochemical findings of all seven cases are shown in Table 2, and representative pathological findings are shown in Figure 1. There were no unique morphological characteristics of the PAL cases. All cases were diagnosed as DLBCL. The tumor cells expressed CD20 (7 of 7), CD79a (6 of 6), BCL2 (5 of 7), BCL6 (4 of 7) and MUM1 (5 of 7). CD10 was negative in all cases. CD5 was positive in two cases. The Ki-67 index was generally high (> 70%). A flow cytometric analysis of the lymphoma cells (data not shown) was available for Cases 6 and 7, and did not conflict with the immunohistochemical data described above. According to Hans’ criteria [10], most of the cases (6 of 7) were classified as the non-germinal center B-cell subtype.

**Table 2:** Summary of pathological and immunohistochemical findings

**Case**	**Pathological diagnosis**	**CD5**	**CD10**	**CD20**	**CD79a**	**BCL2**	**BCL6**	**EBER**	**MUM1**	**Ki-67 index**	**Phenotype**
1	DLBCL, NOS	−	−	+	+	+	−	+	−	70-80%	Non-GCB
2	DLBCL, NOS	−	−	+	+	−	+	−	−	90%	GCB
3	DLBCL, NOS	+	−	+	+	+	−	−	+	80%	Non-GCB
4	DLBCL, NOS	−	−	+	ND	+	+	−	+	75-90%	Non-GCB
5	DLBCL, NOS	+	−	+	+	+	+	−	+	90-99%	Non-GCB
6	DLBCL, NOS	−	−	+	+	+	+	−	+	75-90%	Non-GCB
7	DLBCL, NOS	−	−	+	+	−	−	−	+	90-99%	Non-GCB

*DLBCL* diffuse large B-cell lymphoma, *GCB* germinal center B-cell phenotype, *ND* not done, *NOS* not otherwise specified.

### Treatment and outcomes

All patients were treated with rituximab-containing chemotherapy, such as cyclophosphamide, doxorubicin, vincristine, prednisolone and rituximab (R-CHOP), with a successful clinical outcome (Table 3). The courses of chemotherapy and the doses of agents were modified at the discretion of the attending physicians according to the clinical condition of the patients. In Case 4, because of the apparent coexistence of CNS lymphoma, combined immunochemotherapy plus whole-brain radiotherapy (WBRT), high-dose methotrexate (HD-MTX), high-dose cytarabine and rituximab, was performed according to a previous report [11]. Case 5, in which progression toward the CNS could not be excluded, received systemic intravenous administration of high-dose methotrexate. Two additional cases (Cases 2 and 6) received prophylactic intrathecal injection of methotrexate.

**Table 3:** Summary of clinical courses

**Case**	**Treatment**	**Response to treatment**	**Overall survival duration (months)**	**Relapse**	**Outcome**	**Cause of death**
1	R-CHOP ×5	PR	45.0	No	Dead	Cholangiocarcinoma
	(75% dose)					
2	R-CHOP ×6	PR	7.9	No	Dead	Pneumonia
	IT*					
3	R-CHOP ×2	NA	4.0	−	Dead	Exacerbation of pulmonary fibrosis
	(70% dose)					
4	R-MPV ×4	CR	40.1	No	Alive	−
	IT*					
	HD-Ara-C ×2					
	WBRT					
5	R-CHOP ×6	CR	28.0	No	Alive	−
	HD-MTX ×2					
	IT*					
6	R-CHOP ×8	CR	24.8	No	Alive	−
	IT*					
7	R-COP ×1	NA	2.6	−	Dead	Pneumonia
	(60% dose)					

*IT represents prophylactic administration of methotrexate and/or cytarabine.

*CR* complete response, *CRu* unconfirmed complete response, *HD-Ara-C* high-dose cytarabine, *HD-MTX* high-dose methotrexate, *IT* intrathecal themotherapy, *NA* not assessable, *R-CHOP* rituximab, cyclophosphamide, doxorubicin, vincristine, and prednisolone, *R-COP* rituximab, cyclophosphamide, vincristine, and prednisolone, *R-MPV* rituximab, high-dose methotrexate, procarbazine, and vincristine, *WBRT* Whole-brain radiotherapy.

After the completion of chemotherapy, we assessed the response in each case. CT scans were performed in all cases, and FDG-PET was also performed in some cases (Cases 4, 5 and 6). In the five patients who received a full course of rituximab-containing chemotherapy and the additional therapy mentioned above, a favorable clinical outcome (complete response in Cases 4, 5 and 6, and partial responses in Cases 1 and 2) was achieved, and there have been no cases of relapse as of the writing of this manuscript. The other two patients died of complications during the course of chemotherapy (Case 3, exacerbation of pulmonary fibrosis; Case 7, pneumonia). In both cases, no signs of lymphoma progression, such as further elevation of the serum LDH level, were detected within their clinical course after the initiation of chemotherapy.

**Figure 2:**
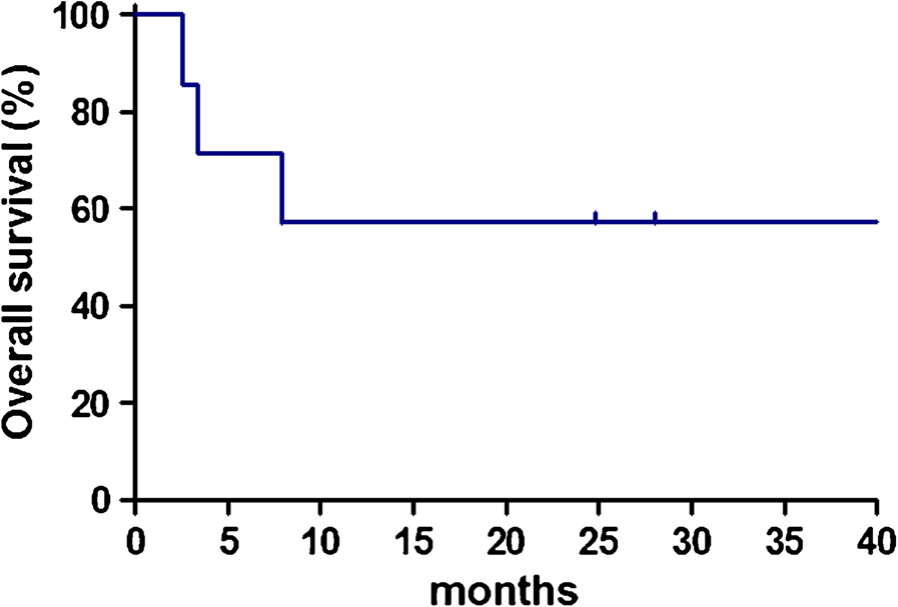
The overall survival in all seven cases of PAL.

The median survival time of all patients from diagnosis until death or the date of the last follow-up was 24.8 months (range, 2.6 – 45.0 months). No patients experienced a relapse of lymphoma, and none died due to the progression of lymphoma. Both the OS and FFS at two years were 57.1% (95% CI 17.2 – 83.7, Figure 2).

## Discussion

PAL is an extremely rare disease entity and only about 100 cases have been reported in the English literature to date. The characteristics of our series of were largely consistent with the previous reports; all were pathologically diagnosed as DLBCL, and the patients were predominantly elderly males [1-4]. It might be unique that two cases were positive for CD5 in our case series; for CD5-positive primary adrenal DLBCL cases have scarcely been reported and only one case was reported recently [12]. However, CD5 expression has not been evaluated even in some recent studies which included a relatively large number of primary adrenal DLBCL cases, and the significance of CD5 expression in primary adrenal DLBCL remains to be known. Three patients were asymptomatic at presentation, and they were incidentally suspected to have adrenal tumors based on CT scans performed as a screening for another disease (i.e., incidentalomas). These observations suggested that PAL might be more prevalent than has been reported.

The definition of primary adrenal lymphoma has not been made clear in the literature, unlike other rare extranodal lymphomas, such as primary bone lymphoma [13] or primary breast lymphoma [14]. A common feature of the definition of the two diseases mentioned above is that the lymphoma arises in a specific extranodal organ, with or without adjacent lymph node swelling. It is thought to be reasonable that this definition is also applicable to primary adrenal lymphoma (PAL). In our cases which had paraaortic lymph node lesions (Cases 3, 5, 6 and 7), the size of the adrenal tumors was larger than that of the lymph node lesions on CT (Table 1). Images of PET-CT scans showed that the SUVmax of the adjacent lymph node lesions was also lower than that of the adrenal lesions (Table 1). These findings suggested that the adrenal lesion was the primary tumor in each case. In Case 4, cerebral and adrenal lesions were detected simultaneously. It is reported that DLBCL with adrenal involvement could have a tendency to develop CNS infiltration [15], so we think that simultaneous involvement of CNS and adrenal glands by lymphoma would be considered as PAL with secondary CNS involvement, as Rashidi A et al. recently stated [16].

The most common symptoms of PAL at presentation were reported to be abdominal or back pain, fever of unknown origin and signs of adrenal insufficiency [1-4]. Bilateral adrenal involvement of PAL is frequently accompanied by clinical adrenal insufficiency [3,4,17-22]. In our series, clinically definite adrenal insufficiency was seen in only one of the five cases in whom the adrenal function was examined. The serum cortisol levels at baseline were not reduced in any of these cases. Partial adrenal insufficiency, which was asymptomatic and detected by the rapid ACTH test, was established in two cases of bilateral adrenal involvement. Lymphoma cells show diffuse infiltration into the adrenal parenchyma, and it is reasonable that bilateral involvement of lymphoma would lead to a condition of adrenal insufficiency, even if it is not clinically obvious.

Gene expression studies of DLBCL have identified two subtypes; the germinal-center B-cell-like (GCB) subtype and the non-GCB subtype (combining the activated B-cell-like subtype and the type 3 subtype) [23]; the latter are associated with a significantly lower survival than the former. Moreover, Li et al. recently showed that non-GCB DLBCL cases with high Ki-67 expression, which is an index of proliferation, received a limited survival benefit from standard R-CHOP therapy [24]. Following the Hans’ algorithm [10], we confirmed that most of our cases were classified as the non-GCB subtype, and all had high Ki-67 expression (over 70%). In other previous reports [1,7], most PAL cases were subclassified as the non-GCB type; these findings may be associated with the poor prognosis of PAL.

In addition to the pathological factors mentioned above, some clinical factors for predicting the prognosis of DLBCL have recently been reported. For example, a high sIL-2R level was reported to be associated with a poor prognosis among patients with DLBCL who were treated with R-CHOP [25]. It has also been reported that a high SUVmax on PET scans is associated with a shorter survival in patients with DLBCL [26]. Moreover, Shou *et al.* recently reported that lymphomas showing intense FDG uptake have an increased capacity for proliferation and rapid growth, which was confirmed by the Ki-67 expression [27]. In the present study, the bilateral cases had high levels of sIL-2R and a high SUVmax on PET/CT compared with unilateral cases. The Ki-67 index was also generally high in all cases. These points suggest that bilateral adrenal DLBCL should be a clinically aggressive disease.

We successfully treated PAL patients in a tolerable general condition with rituximab-containing systemic chemotherapy and CNS prophylaxis. PAL is frequently treated with cyclophosphamide, doxorubicin hydrochloride, vincristine and prednisolone (CHOP), or a CHOP-like regimen; however, the prognosis is generally poor [1,2]. In recent years, rituximab has been added to the regimen, and there have been a few reports about successful outcomes with rituximab-containing regimens for PAL [5,28,29]. However, CNS relapses of PAL occur frequently, and most patients with CNS relapse are reported to have died early [3,4,19,30,31]. We treated most of the patients in the present study by R-CHOP, with modifications as necessary, and added HD-MTX and/or intrathecal chemotherapy for the patients who were considered to potentially have CNS infiltration at presentation. It is noteworthy that all of the patients who received a full course of rituximab-containing chemotherapy and CNS-oriented treatment are currently alive without disease progression or relapse. No patients died of the progression of lymphoma, and there were no cases of CNS relapse. This is despite the non-GCB DLBCL subtype, elevated sIL-2R level and Ki-67 index noted in these patients. Therefore, in younger PAL patients in good general condition, an aggressive chemotherapeutic strategy consisting of a rituximab-containing chemotherapy regimen and CNS-oriented treatment is thought to be promising, and could improve the prognosis of PAL.

## Conclusions

PAL with DLBCL histology is a clinically aggressive disease. Rituximab-containing chemotherapy combined with CNS prophylaxis could be a reasonable option for the treatment of PAL; however, further accumulation of clinical experiences, as well as additional research, is necessary to establish the optimal therapeutic strategy for PAL.

## Methods

### Case selection and clinical data

From 2008 to 2011 in our institutions, a total of 563 patients were diagnosed with DLBCL. We reviewed the records of the patients with newly diagnosed primary adrenal DLBCL. Cases with secondary adrenal involvement and those with human immunodeficiency virus infection were excluded. A total of seven cases were included in this study. All had unilateral or bilateral adrenal tumors, which were the predominant tumors considered to be primary lesions (and were biopsied), while some other lesions, such as paraaortic lymph node swelling or central nervous system lesions, were also detected in several cases. The therapeutic strategy was chosen at the discretion of the attending physicians.

All available data, including the clinical presentations, laboratory findings, radiological findings, therapeutic regimens and follow-up information, were evaluated. The staging procedures included a complete physical examination, computed tomography (CT) scans of the neck, chest and abdomen, positron emission tomography (PET) scans and bone marrow biopsy and/or aspiration. This study was approved by the Ethics Committee of each hospital. The procedures performed for the present study were done in accordance with the Declaration of Helsinki.

### Pathological evaluation

Tissue specimens were fixed in 10% formalin solution, routinely processed and embedded in paraffin. The sections were stained with hematoxylin-eosin for microscopic examination. Immunohistochemical studies were performed using antibodies against CD3, CD5, CD10, CD20, CD45RA, CD79a, BCL2, BCL6, MUM1, Ki-67 and Epstein–Barr virus-encoded small RNA (EBER). For two cases with sufficient materials available, flow cytometry was performed using various monoclonal antibodies against CD2, CD3, CD4, CD5, CD7, CD8, CD10, CD13, CD19, CD20, CD22, CD30, CD45, CD56, TCRαβ, TCRγδ, Igκ and Igλ. Markers that were expressed on more than 20% of the cells were judged to be positive.

### Statistical analysis

All statistical analyses were performed using the GraphPad Prism software program (version 5.04 for Windows; GraphPad Software Inc., San Diego, CA). The treatment outcomes were measured by the failure-free survival (FFS) and overall survival (OS). The FFS was defined as the time from initial diagnosis to the first occurrence of progression, relapse after a response or death from any cause. The follow-up of patients not experiencing one of these events was censored at the date of last contact. The OS was measured from the time of the initial diagnosis until death from any cause, with surviving patient follow-up censored at the last contact date. Estimates of the FFS and OS were determined using the Kaplan–Meier method.

## Competing interests

The authors declare no competing interests.

## Authors’ contributions

SI, the first author, designed the research project, analyzed the data, reviewed the literature, drafted the manuscript and revised the final manuscript. NF, the corresponding author, designed the research project, contributed to discussions and reviewed the manuscript. AI, RO, YK, YO, JY, OS, JN and OF were in charge of the treatment and follow-up of the patients and collected the patient information. HK interpreted the pathological slides. KI contributed to the discussion and review of the manuscript. RI interpreted the pathological slides and contributed to the discussion and review of the manuscript. HH, the last author, supervised the overall project and contributed to the discussion and review of the manuscript. All authors reviewed and approved the final version of the manuscript.
